# MbnC Is Not Required for the Formation of the N-Terminal Oxazolone in the Methanobactin from Methylosinus trichosporium OB3b

**DOI:** 10.1128/AEM.01841-21

**Published:** 2022-01-25

**Authors:** Philip Dershwitz, Wenyu Gu, Julien Roche, Christina S. Kang-Yun, Jeremy D. Semrau, Thomas A. Bobik, Bruce Fulton, Hans Zischka, Alan A. DiSpirito

**Affiliations:** a Roy J. Carver Department of Biochemistry, Biophysics and Molecular Biology, Iowa State University, Ames, Iowa, USA; b Department of Civil and Environmental Engineering, University of Michigan, Ann Arbor, Michigan, USA; c Institute of Molecular Toxicology and Pharmacology, Helmholtz Center Munich, German Research Center for Environmental Health, Neuherberg, Germany; d Technical University Münich, School of Medicine, Institute of Toxicology and Environmental Hygiene, Munich, Germany; North Carolina State University

**Keywords:** methanobactin, chalkophore, methanotroph, aerobic methane oxidation, ribosomally synthesized and posttranslational modified peptide

## Abstract

Methanobactins (MBs) are ribosomally synthesized and posttranslationally modified peptides (RiPPs) produced by methanotrophs for copper uptake. The posttranslational modification that defines MBs is the formation of two heterocyclic groups with associated thioamines from X-Cys dipeptide sequences. Both heterocyclic groups in the MB from Methylosinus trichosporium OB3b (MB-OB3b) are oxazolone groups. The precursor gene for MB-OB3b is *mbnA*, which is part of a gene cluster that contains both annotated and unannotated genes. One of those unannotated genes, *mbnC*, is found in all MB operons and, in conjunction with *mbnB*, is reported to be involved in the formation of both heterocyclic groups in all MBs. To determine the function of *mbnC*, a deletion mutation was constructed in *M. trichosporium* OB3b, and the MB produced from the Δ*mbnC* mutant was purified and structurally characterized by UV-visible absorption spectroscopy, mass spectrometry, and solution nuclear magnetic resonance (NMR) spectroscopy. MB-OB3b from the Δ*mbnC* mutant was missing the C-terminal Met and was also found to contain a Pro and a Cys in place of the pyrrolidinyl-oxazolone-thioamide group. These results demonstrate MbnC is required for the formation of the C-terminal pyrrolidinyl-oxazolone-thioamide group from the Pro-Cys dipeptide, but not for the formation of the N-terminal 3-methylbutanol-oxazolone-thioamide group from the N-terminal dipeptide Leu-Cys.

**IMPORTANCE** A number of environmental and medical applications have been proposed for MBs, including bioremediation of toxic metals and nanoparticle formation, as well as the treatment of copper- and iron-related diseases. However, before MBs can be modified and optimized for any specific application, the biosynthetic pathway for MB production must be defined. The discovery that *mbnC* is involved in the formation of the C-terminal oxazolone group with associated thioamide but not for the formation of the N-terminal oxazolone group with associated thioamide in *M. trichosporium* OB3b suggests the enzymes responsible for posttranslational modification(s) of the two oxazolone groups are not identical.

## INTRODUCTION

Methanobactins (MBs) are low-molecular-mass (<1,300 Da), posttranslationally modified copper-binding peptides excreted by some methanotrophs as the extracellular component of a copper acquisition system (1–7). Structurally MBs are characterized by the presence of a C-terminal oxazolone group with a C2-associated thioamide and by the presence of an N-terminal oxazolone, imidazolone or pyrazinedione group with an associated thioamide. Some MBs also contain a sulfate group in-place of the hydroxyl group on a Tyr adjacent to the C-terminal oxazolone group. The best-characterized MB is from Methylosinus trichosporium OB3b (MB-OB3b), and the posttranslational modifications for this MB involve (i) deamination of the N-terminal Leu, (ii) conversion of the N-terminal Leu-Cys dipeptide to 1-(*N*-(mercapto-(5-oxo-2-(3-methylbutanoyl)oxazol-(*Z*)-4-ylidene)methyl), (iii) conversion of the C-terminal Pro-Cys dipeptide into pyrrolidin-2-yl-(mercapto-(5-oxo-oxazol-(*Z*)-4-ylidene)methyl), and (iv) cleavage of the leader sequence (2, 4, 5, 8–11).

The gene encoding the MB precursor peptide, *mbnA* (5, 10), is found in a gene cluster that contains both genes of known function, such as *mbnB* (5, 11), *mbnN* (9), and *mbnT* (12), as well as unannotated genes, such as *mbnC* (5, 10, 11, 13, 14). MbnB is a member of TIM barrel family as well as the DUF692 family of diiron enzymes (11, 14). In heterologous expression studies in Escherichia coli, MbnBC was shown to catalyze a dioxygen-dependent four-electron oxidation of Pro-Cys in MbnA (11, 14, 15). The roles of MbnB and MbnC could not be separately determined as attempts to separately purify these gene products in E. coli failed (11). From these data, it has been argued that MbnBC must act in concert and by doing so create both heterocyclic groups in MBs (11). Such conclusions, however, appear to be premature for several reasons. First, the reported spectra (11) only show the presence of the C-terminal oxazolone group, not the N-terminal oxazolone group, as the 394-nm absorption maximum is missing. Second, the absorption maximum at 302 nm, diagnostic for the presence of the N-terminal oxazolone group, was absent (5, 8, 16). Third, no structural data were provided to support the presence of both oxazolone groups. To examine if MbnB and -C act in concert and are involved in the formation of both oxazolone groups in *M. trichosporium* OB3b, an MnbC deletion mutant (Δ*mbnC*) was constructed. The results show MbnC is required for the formation of the C-terminal oxazolone group, but not for the formation of the N-terminal oxazolone group.

## RESULTS

### Generation of the Δ*mbnC* mutant.

The previously constructed Δ*mbnAN* strain, whereby the *mbnABCMN* genes were deleted using a sucrose counterselection technique (9), was back complemented with *mbnABMN* through selective amplification and ligation of *mbnAB* with *mbnMN*, deleting *mbnC*, and inserting this ligation product into pTJS140, creating pWG104 (Table 1). Successful removal of *mbnC* from this product was confirmed via sequencing (data not shown). The native σ^70^-dependent promoter upstream of *mbnA* was also incorporated into pWG104, and expression of *mbnABMN* but not *mbnC* (from pWG104), as well as *mbnPH* (from the chromosome) was confirmed via reverse transcription-PCR (RT-PCR) (see Fig. S1 and S2 in the supplemental material).

**TABLE 1:** Strains, plasmids, and primers used in this study

Strain, plasmid, or primer	Description*^a^*	Restriction site	Reference or source
Strains			
Escherichia coli			
TOP10	F^−^ *mcrA* Δ(*mrr*-*hsdRMS*-*mcrBC*) ϕ80*lacZ*ΔM15 Δ*lacX74 recA1 araD139* Δ(*ara leu*)*7697 galU galK rpsL* (Str^r^) *endA1 nupG*		Invitrogen
S17-1 *λpir*	*recA1 thi pro hsdR* mutant RP4-2Tc::Mu Km::Tn*7* λ*pir*		26
Methylosinus trichosporium			
OB3b	Wild-type strain		
Δ*mbnAN* mutant	*mbnABCMN* deleted		9
Δ*mbnC* mutant	Δ*mbnAN* carrying pWG104		this study

Plasmids			
pTJS140	Broad-host-range cloning vector; Mob Ap^r^ Sp^r^ Sm^r^ *lacZ*		34
pWG104	pTJS140 carrying *mbnABMN* with its native promoter		This study

Primers			
mbnANf	ATTTTTggtaccGACGTTCGGGTCTTCTTCGC	KpnI	9
mbnANr	ATTTTTggtaccCGCCTCTAGATCATTCCGAC	KpnI	9
mbn66	ATTTTTggatccCGAACAATGTGTGCCAGTAG	BamHI	This study
mbn70	ATTTTTggatccGTTCGGCTATTTCCTGACGC	BamHI	This study
qmbnA_FO	TGGAAACTCCCTTAGGAGGAA		35
qmbnA_RO	CTGCACGGATAGCACGAAC		35
qmbnB_F1	TGGTCCAGCAGATGATCAAAG		This study
qmbnB_R2	TTCCCGAGCTTCTCCAATTC		This study
dmbnC_F	GGGAGAACAACCTCGCTTT		This study
dmbnC_R	CTTCCCAGCACGATCTGAC		This study
qmbnM_F	GCTAGGCTGGCTCCTTTATC		This study
qmbnM_R	GATGTTGACCACAAACCGAAAG		This study
qmbnN_F	CGATTCCATCCTTTCCGATGT		This study
qmbnN_R	CACTTTCGAAGACAAGGAGAGA		This study
qmbnP_F	AAAGGGAAGCACACACCCAT		This study
qmbnP_R	GTCGTGTTCTTGGCCGGATT		This study
qmbnH_F	ACTTACCGAAATACATCCCGC		This study
qmbnH_R	CGGAGAGGCGCTTATCGTAG		This study

a Added overhangs for binding by restriction enzyme are underlined. Restriction sites are noted with lowercase letters.

### UV-visible absorption and mass spectrometry of metal-free MB from *M. trichosporium* OB3b *ΔmbnC*.

**FIG 1:**
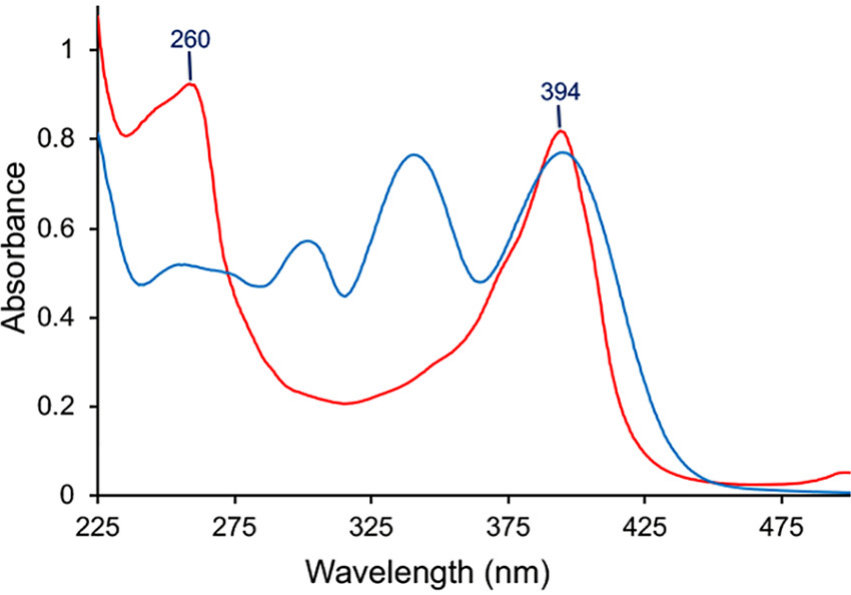
UV-visible absorption spectra of MB-OB3b (blue) and the Δ*mbnC* mutant (red). Abbreviations: OxaA, oxazolone A or the N-terminal oxazolone group; OxaB, oxazolone B or the C-terminal oxazolone group.

**FIG 2:**
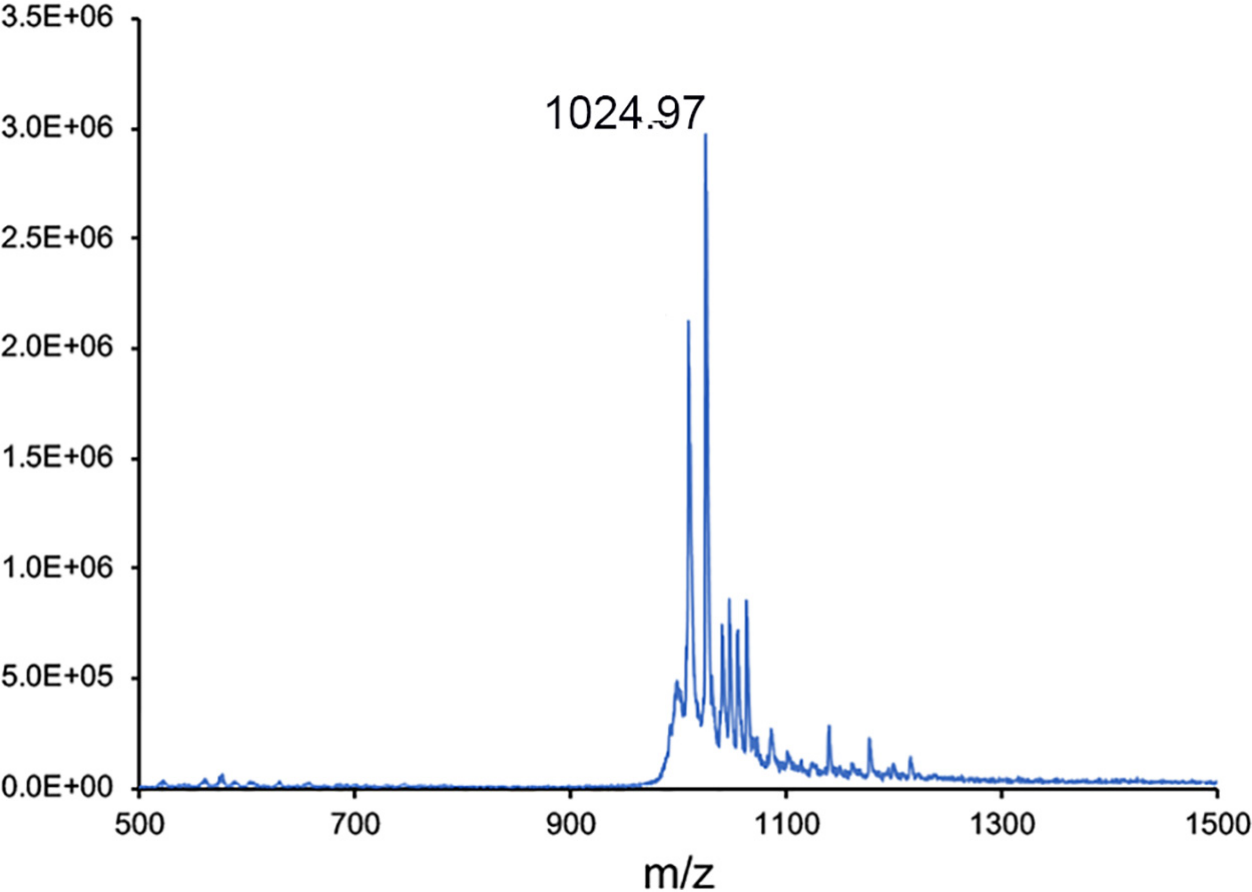
MALDI-TOF MS of methanobactin from Δ*mbnC*.

**FIG 3:**
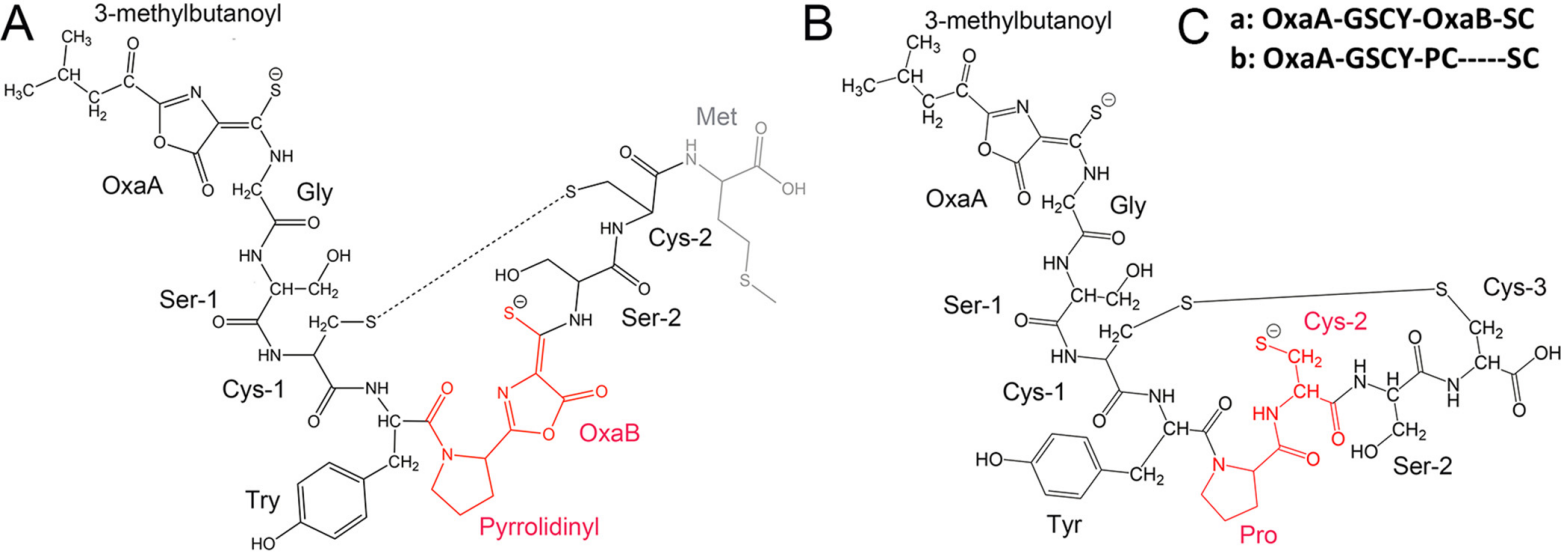
(A) Structure of wild-type MB-OB3b, with the labile terminal methionine in gray. (B) Proposed structure of the Δ*mbnC* mutant based on UV-visible absorption spectra, LC-MS, and NMR analysis. The differences between MB-OB3b-Met and the Δ*mbnC* mutant are highlighted in red. (C) Amino acid sequence of (a) wild-type MB-OB3b minus the C-terminal Met and (b) the Δ*mbnC* mutant.

Comparison of the UV-visible absorption spectra of MB from *M. trichosporium* OB3b *ΔmbnC* to wild-type MB-OB3b suggested the of presence of the N-terminal oxazolone group, but the absence of C-terminal oxazolone (Fig. 1: see Fig. S3 in the supplemental material). The molecular mass of native, full-length MB-OB3b is 1,154 Da, and that of MB-OB3b lacking the C-terminal Met is 1,023 Da. It should be noted that both forms of MB-OB3b are present in most MB-OB3b preparations (2, 5, 17). The molecular mass of ΔMbnC was 1,024 as determined by matrix-assisted laser desorption ionization-time of flight (MALDI-TOF) mass spectrometry (MS) (Fig. 2) (9), which was within 1 Da of the predicted molecular mass of MB-OB3b, in which only one oxazolone group was formed. Taken together, the UV-visible absorption spectra and molecular mass data suggest the Δ*mbn*C mutant lacked the C-terminal Met as well as the N-terminal oxazolone group with a 1-(*N*-[mercapto-(5-oxo-2-(3-methylbutanoyl)oxazol-(*Z*)-4-ylidene)methyl]-GSCYPCSC predicted structure (Fig. 3B). In contrast to wild-type MB-OB3b, the C-terminal Met was never observed in MbnC.

### Chemical structure of metal-free Δ*mbnC* mutant as determined by NMR spectroscopy.

Metal-free MB has multiple conformations, making structural studies of MBs via solution nuclear magnetic resonance (NMR) or crystallography difficult (see Fig. S4 in the supplemental material). In prior structural studies of MB, the addition of Cu^2+^ (which is bound and reduced to Cu^1+^ by native MB-OB3b) stabilizes MB-OB3b into one conformation, allowing for crystal formation and NMR characterization (Fig. S4) (2–5, 8, 18). Our initial efforts to investigate the structure of the MB intermediate produced by the Δ*mbnC* strain via NMR were unsuccessful. In contrast to native MB, the MB intermediate from the Δ*mbnC* strain bound, but did not reduce Cu^2+^ to Cu^+^, resulting in peak broadening from paramagnetic Cu^2+^. This necessitated a different strategy. Substituting other metals with similar binding behavior for copper such as Au^3+^, Zn^2+^, Co^2+^, and Ni^2+^ also failed to produce well-behaved complexes. Therefore, it was necessary to examine the metal-free Δ*mbnC* mutant.

**FIG 4:**
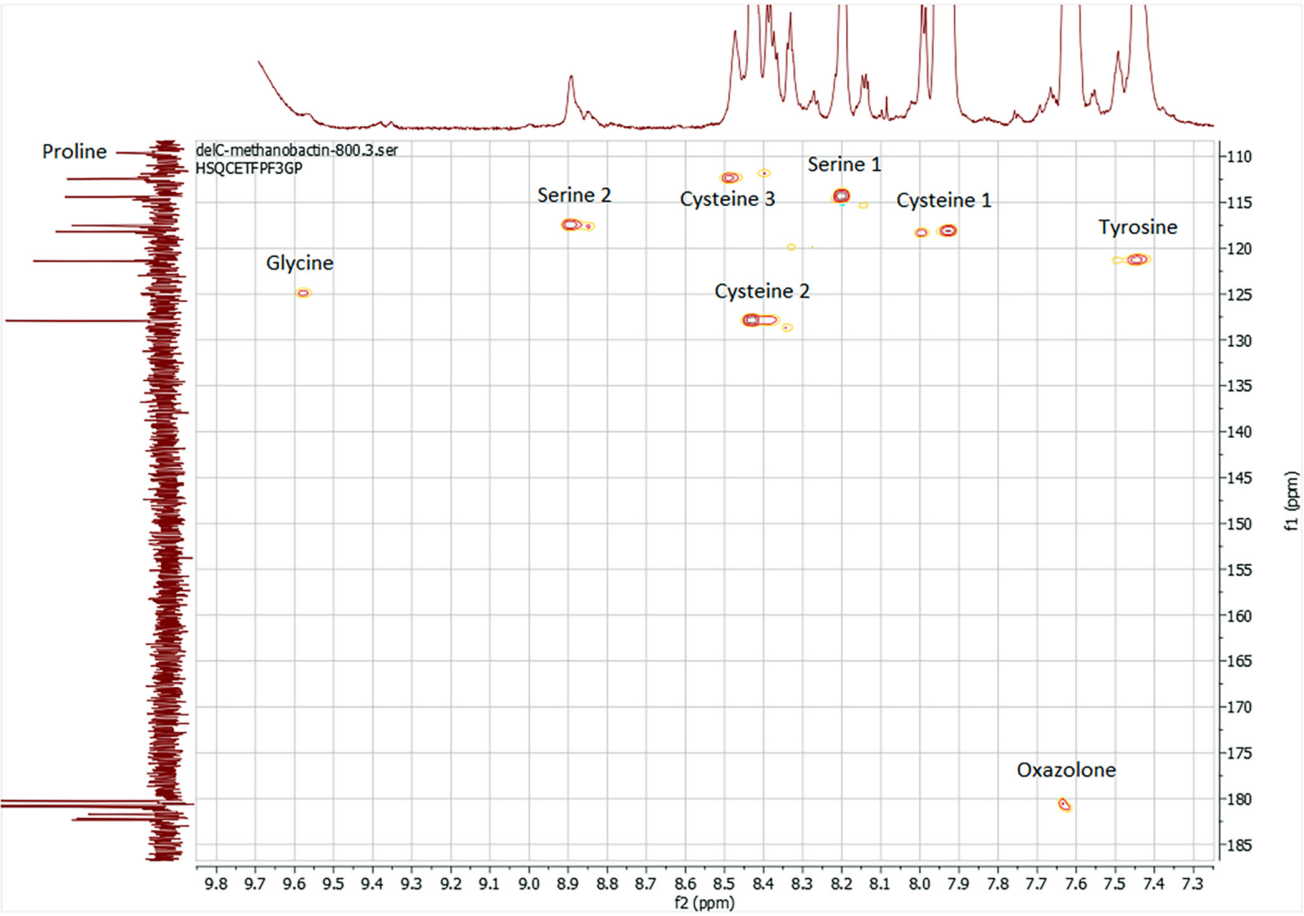
The 800-MHz ^1^H-^15^N-HSQC spectrum of uniformly ^15^N-labeled Δ*mbnC* mutant in 90% 9 mM phosphate buffer (pH 6.5) and 10% D_2_O at 265 K and 300,000,000 Pa. The horizontal and vertical 1D spectra are ^1^H and ^15^N spectra, respectively.

At standard temperature and pressure, the Δ*mbnC* mutant undergoes exchange between multiple conformations on an intermediate time scale, leading to excessive line broadening (see Fig. S5 in the supplemental material). In order to slow down the rate of exchange and reduce line broadening, we sampled various temperature and hydrostatic pressure conditions. We found that two-dimensional (2D) ^1^H-^15^N NMR spectra of the Δ*mbnC* mutant recorded at high pressure (300,000,000 Pa) and low temperature (265 K) (18, 19) show significantly reduced line broadening and gave excellent spectra in the absence of copper (Fig. 4).

A series of NMR experiments were conducted on the Δ*mbnC* mutant, including homonuclear correlation spectroscopy (COSY), total correlation spectroscopy (TOCSY), rotating-frame nuclear Overhauser effect spectroscopy (ROESY), ^1^H-^15^N and ^1^H-^13^C heteronuclear single-quantum correlation spectroscopy (HSQC), and heteronuclear multiple-bond correlation spectroscopy (HMBC). These experiments enabled assigning all nonhydroxyl ^1^H, nonconjugated ^13^C, and all ^15^N resonances (Table 2 and Fig. 4; see Fig. S6 in the supplemental material). The assigned chemical shifts show that the MB from the Δ*mbnC* mutant contains 8 amino acids—3 Cys, 2 Ser, 1 Gly, 1 Tyr and 1 Pro—and 1 oxazolone group (Fig. 4). The 1D ^15^N experiment showed a peak at 109 ppm that was absent from the ^1^H-^15^N HSQC spectra and was assigned to proline. However, the glycine nitrogen peak was especially broad, and could only be assigned with the ^1^H-^15^N HSQC. Finally, while the 1D ^15^N experiment had several resonances around 180 ppm—likely due to hydrolysis and deprotonation—only one of them had a correlation with ^1^H in the ^1^H-^15^N HSQC, indicating a single oxazolone group. The NMR results are consistent with the UV-visible absorption spectra and the ESI MS results, as well as with the structure shown in Fig. 3B.

**TABLE 2:** ^1^H, ^13^C, and ^15^N resonances for metal-free Δ*mbnC* mutant^*a*^

Residue	Atom	Chemical shift (ppm)			Residue	Atom	Chemical shift (ppm)		
		^1^H	^13^C	^15^N			^1^H	^13^C	^15^N
3-Methyl-butanoyl	C^1^		174.6		Tyr^4^	H^N^	7.44		
	C^2^		50.5			H^α^	2.96		
	C^3^		38.0			H^β^	2.79		
	C^4^		19.6			H^β^	1.20		
	C^5^		19.6			H^2,6^	6.11		
	H^2^	4.15				H^3,5^	6.45		
	H^3^	2.17			Pro^5^	N^1^			109.6
	H^3^	2.72				C^2^		67.3	
	H^4^	1.88				C^3^		21.1	
	H^5^	1.80				C^4^		39.5	
Oxazolone	N			180.1		C^5^		55.2	
	H^N^	7.61				H^2^	3.67		
Gly^1^	N			125.1		H^3^	1.06		
	C					H^3^	2.13		
	C^α^		26.6			H^4^	1.28		
	H^N^	9.57				H^4^	2.29		
	H^α^	1.46				H^5^	2.79		
Ser^2^	N			114.3		H^5^	2.96		
	C		181.6		Cys^6^	N			127.9
	C^α^		72			C		136.3	
	C^β^					C^α^		53.3	
	H^N^	8.19				C^β^		49.3	
	H^α^	4.14				H^N^	8.43		
	H^β^	3.98				H^α^	3.96		
	H^β^	1.41				H^β^	3.23		
Cys^3^	N			118.1		H^β^	1.38		
	C		173.0		Ser^7^	N			117.5
	C^α^		71.2			C			
	C^β^		35.6			C^α^		51.6	
	H^N^	7.93				C^β^		45.0	
	H^α^	3.96				H^N^	8.90		
	H^β^	3.23				H^α^	4.19		
	H^β^	1.37				H^β^	3.25		
Tyr^4^	N			121.5		H^β^	1.48		
	C				Cys^8^	N			112.4
	C^α^		48.9			C		172.6	
	C^β^		35.6			C^α^		42.3	
	C^1^					C^β^		21.1	
	C^2,6^					H^N^	8.47		
	C^3,5^		135.4			H^α^	3.69		
	C^4^					H^β^	3.55		
						H^β^	0.97		

a The table is presented in a two-column format (i.e., the table’s two “Residue” columns and corresponding data are independent from one another).

## DISCUSSION

Due to the variability in the core sequences of structurally characterized MBs, it is difficult to use *mbnA* to screen the potential ability of microbes to produce MB. Instead, *mbnB* and *mbn*C sequences are commonly used as they are found in all known *mbn* gene clusters (5, 13). All known MBs contain two heterocyclic rings, with the N-terminal ring found to be either an oxazolone, pyrazinedione, or imidazolone ring, while the C-terminal ring was always found to be an oxazolone. Given these data, it could be presumed that MbnBC is involved in the formation of the C-terminal oxazolone group along with an associated thioamide, while the N-terminal oxazolone group is formed via a different process, such as the involvement of an aminotransferase, as concluded earlier (5, 9, 10, 13).

Other researchers have attempted to elucidate the role of MbnB and MbnC in methanobactin maturation (11). These individuals were unable to separately heterologously express soluble protein from either MbnB or MbnC, but were able to coheterologously express MbnBC as a heterodimeric complex. In studies where the MbnA precursor polypeptide was incubated with this MbnBC complex, the authors conclude that MbnBC was involved in the formation of both oxazolone groups and the associated thioamides of MB-OB3b. It should be noted, however, that in this study, no structural evidence (i.e., solution NMR data) was provided to definitively show the presence of either ring: rather, such conclusions were largely based on mass spectral analyses of MbnA after incubation with the MbnBC complex. Further, the authors assumed that since their construct did not contain the N-terminal aminotransferase MbnN, the extended conjugation resulting from this reaction would result in both oxazolone groups having identical absorption maxima. The idea that the extended conjugation of the N-terminal oxazolone could be responsible for the bathochromic shift was first proposed as a possible reason for the 50-nm shift in the absorption maxima by Krentz et al. (5). Kenney et al. used this theory to bolster their claim that both oxazolone groups were present in the product from their heterologous system, with both oxazolone groups showing identical absorption spectra (11). The evidence to support this claim came from their *M. trichosporium* OB3b Δ*mbnN* strain. MbnN is responsible for the deamination of the N-terminal Leu in *M. trichosporium* OB3b, extending the conjugation one additional double bond. In this study, the authors claim they can stabilize the MB produced by the Δ*mbnN* strain by the addition of copper before purification. UV-visible absorption spectra of copper-containing Δ*mbnN* mutant suggest the possible presence of two oxazolone groups but no additional evidence was provided supporting this claim.

This observation was surprising as the MB produced by the Δ*mbnN* strain in our laboratory showed similar UV-visible absorption spectra throughout the growth cycle, suggesting the absence of the N-terminal oxazolone group (see Fig. S7 in the supplemental material). In addition, the UV-visible absorption spectra, liquid chromatography (LC)-MS/MS, Fourier transform ion cyclotron resonance (FT-ICR) MS, amino acid analysis, number of thiol groups, copper-binding properties, and pattern of acid hydrolysis demonstrate the absence of the N-terminal oxazolone group in the Δ*mbnN* mutant (9).

Additional evidence that the bathochromic shift in MBs with two oxazolone groups is unlikely to solely arise from the addition of one double bond following deamination of the N-terminal amine comes from examination of the group I MB from Methylocystis parvus OBBP. Acid hydrolysis of the MB from *M. parvus* OBBP shows a similar hydrolysis pattern to that observed with the MB from *M. trichosporium* OB3b, demonstrating the presence of two oxazolone groups, with absorption maxima at 340 and 390 nm (see Fig. S8 in the supplemental material). However, both MB operons from *M. parvus* OBBP lack *mbnN*, and without deamination of the N-terminal Phe, the conjugation around the N-terminal oxazolone group would not be extended. It is possible that another aminotransferase in the *M. parvus* OBBP genome may catalyze deamination of the N-terminal Phe. However, this appears unlikely as deamination of the N-terminal amino acid has never been observed in structurally characterized MBs from operons lacking *mbnN* (3, 5). The results suggest deamination of the N-terminal amino acid is not solely responsible for the 40- to 50-nm absorption maximum difference between oxazolone groups in MBs. The absence of either the N-terminal or C-terminal oxazolone group in a small (0.5 to 2%) fraction of most MB-OB3b preparations (Fig. S3) also questions the suggestion that the absorption maximum difference between the N-terminal and C-terminal oxazolone groups is due solely to extending the conjugation of an additional double bond introduced following the deamination reaction.

**FIG 5:**
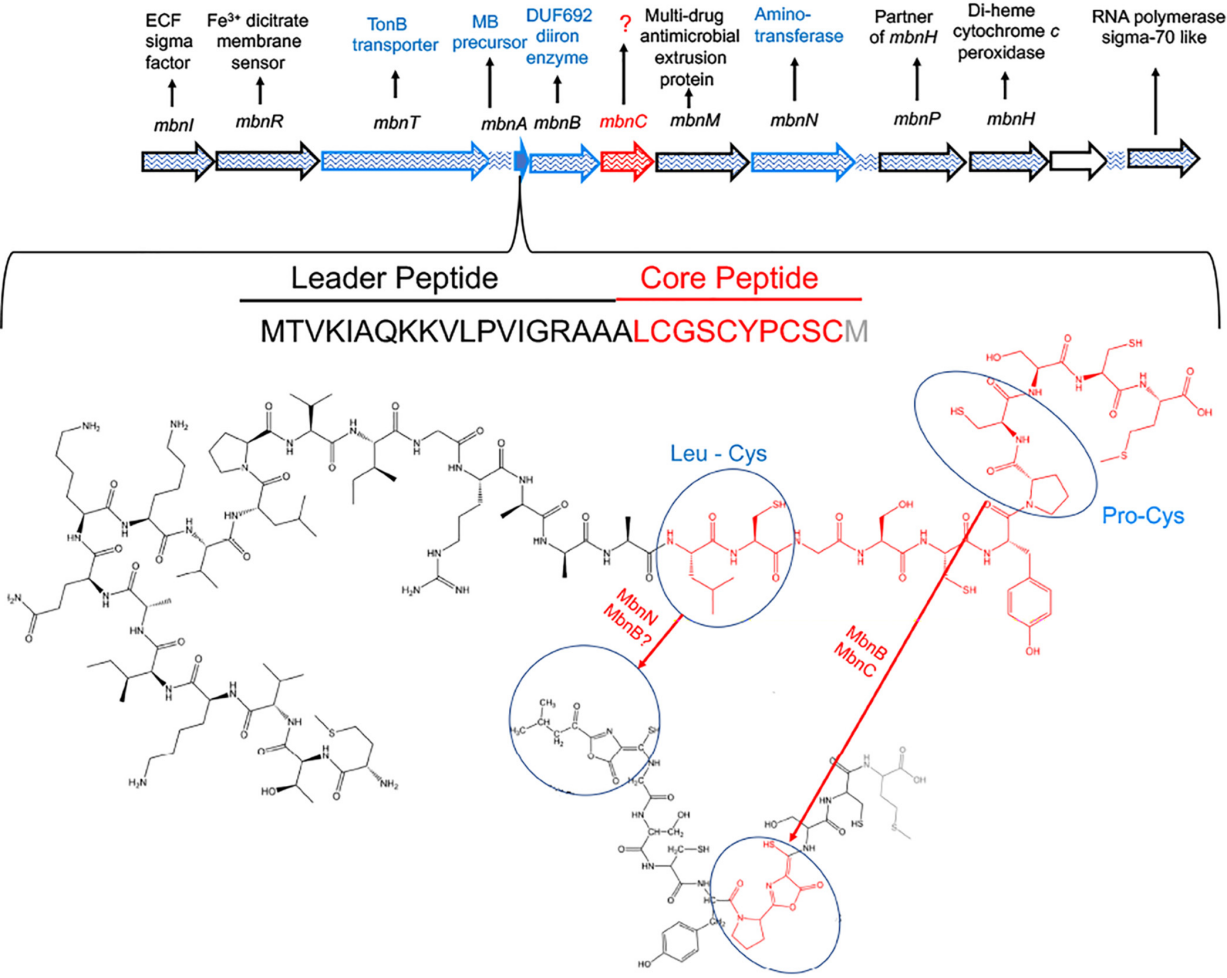
(Top) MB-OB3b gene cluster. Genes with known involvement in MB-OB3b synthesis and transport are shown in blue. (Bottom) Proposed genes involved in the biosynthesis of the oxazolone rings with associated thioamides from MbnA. Additional, yet to be identified genes may also be involved in the formation of oxazolone groups.

The results presented here confirm MbnC is required for the formation of the C-terminal oxazolone group (Fig. 5). However, the results presented here also demonstrate MbnC is not required for the formation of the N-terminal oxazolone group in *M. trichosporium* OB3b, suggesting the formation of the two hetercyclic groups with associated thioamides from XC dipeptides does not utilize the same enzyme(s). Future studies will determine if MbnB is involved in the formation of the N-terminal oxazolone, pyranzinedione, or imidazolone groups. Resolution of the pathway and enzymes responsible for the posttranslational modifications required for the synthesis of MB in methanotrophic bacteria will aid in the production of MB derivatives with pharmacological properties specific for different metal-related diseases (19–24) as well as for environmental applications (10, 25).

## MATERIALS AND METHODS

### Bacterial strains, growth media, and culture conditions.

Plasmid construction was accomplished using Escherichia coli strain TOP10 (Invitrogen, Carlsbad, CA) as described previously (9). Plasmids used and constructed during this study are shown in Table 1. The donor strain for conjugation of plasmids into Methylosinus trichosporium OB3b was E. coli S17-1 (26). E. coli strains were cultivated at 37°C in Luria broth medium (Dot Scientific, Burton, MI). Methanotrophic strains (i.e., *M. trichosporium* OB3b wild type, *M. trichosporium* OB3b Δ*mbnAN*, *M. trichosporium* OB3b Δ*mbnC*, *Methylocystis* sp. strain SB2, and Methylocystis parvus OBBP) were cultivated at 30°C on nitrate mineral salts (NMS) medium (27), either in 250-mL flasks with side-arms at 200 rpm or in a 15-L New Brunswick Bioflow 310 fermenter (Eppendorf, Hauppauge, NY), using methane as the sole carbon and energy source. Where necessary, filter-sterilized solutions of copper (as CuCl_2_) and spectinomycin were added to culture media aseptically. A working concentration of 20 μg mL^−1^ spectinomycin was used for maintaining pWG104 in the *M. trichosporium* OB3b Δ*mbnAN* deletion mutant (i.e., *M. trichosporium* OB3b Δ*mbnC*). Chemicals were purchased from Fisher Scientific (Waltham, MA) or Sigma-Aldrich (St. Louis, MO) of American Chemical Society reagent grade or better.

For ^15^N NMR, K^14^NO_3_ in NMS medium was replaced with K^15^NO_3_ (Cambridge Isotope Laboratories, Cambridge, MA).

### General DNA methods, transformation, and conjugation.

DNA purification and plasmid extraction were performed using QIAquick and QIAprep kits from Qiagen following the manufacturer’s instruction. DNA cloning, preparation of chemically competent cells, and plasmid transformation with E. coli were performed according to reference 28. Enzymes used for restriction digestion and ligation were purchased from New England Biolabs (Ipswich, MA). PCR of DNA for cloning purposes was accomplished using iProof high-fidelity polymerase (Bio-Rad, Hercules, CA). PCR for general purposes was accomplished using GoTaq DNA polymerase (Promega, Fitchburg, WI). PCR programs were set according to the manufacturer’s suggestions. Plasmid pWG104 was conjugated into *M. trichosporium* OB3b Δ*mbnAN* with E. coli S17-1 as the donor strain as described by Martin and Murrell (29).

### Construction of the *M. trichosporium* OB3b Δ*mbnC* strain.

Previously, an *M. trichosporium* mutant was constructed in which *mbnABCMN* was deleted using a counterselection technique (9). To characterize the function of *mbnC*, a Δ*mbnC* mutant was constructed by introducing the pWG104 expression vector into the Δ*mbnAN* mutant. pWG104 was constructed by cloning two separate DNA fragments, one of which was a 1.9-kb DNA fragment of *mbnAB* (created via use of primers mbnANf and mbn66) and the other of which was a 2.5-kb DNA fragment of *mbnMN* (created via use of primers mbn70 and mbnANr), leaving out *mbnC*. These two fragments were amplified with BamHI restriction sites as indicated in Fig. S2. These were then ligated together and cloned into the broad-host-range vector pTJS140 at the KpnI site.

### Extraction of RNA and RT-PCR.

To check the expression of genes restored to the *M. trichosporium* OB3b Δ*mbnC* mutant (e.g., *mbnA*, -*B*, *-M*, and *N*), genes associated with MB remaining in the chromosome (*mbnPH*), as well as the absence of *mbnC*, RNA from the Δ*mbnC* mutant was collected, purified, and reverse transcribed to cDNA to perform RT-PCR. Total RNA was isolated as described earlier (9). Briefly, the Δ*mbnC* mutant was grown to the exponential phase, and RNA was extracted using a phenol-chloroform method modified from Griffiths et al. (30). Collected RNA was purified and removal of DNA confirmed by the absence of 16S rRNA PCR product from PCRs. The same amount of RNA (500 ng) was used for reverse transcription by SuperScript III reverse transcriptase (Invitrogen, Carlsbad, CA) for all reactions. RT-PCR analyses were performed to confirm the expression of *mbnABMNPH* as well as the absence of *mbnC* using primers listed in Table 1.

### Isolation of MB *from M. trichosporium* OB3b, *Methylocystis s*train SB2, Methylocystis parvus OBBP, and the Δ*mbn*C mutant.

MBs from all three methanotrophs were purified as previously described (31).

### UV-visible absorption spectra.

UV-visible absorption spectra of MbnC^−^ high-performance liquid chromatography (HPLC) fractions from MB preparations from *M. trichosporium* OB3b and *Methylocystis* strain SB2 and from the MB from *M. parvus* OBBP were determined as previously described (32, 33). Acid hydrolysis of the oxazolone groups in the MB from *M. parvus* OBBP was carried out in 85 μM acetic acid as previously described (32)

### Structural characterization of the Δ*mbnC* mutant.

UV-visible spectroscopy was recorded on a Cary 50 spectrometer (Agilent, Santa Clara, CA). Matrix-assisted laser desorption ionization-time of flight (MALDI-TOF) MS was performed on a Shimadzu AXIMA Confidence MALDI-TOF mass spectrometer (Shimadzu, Kyoto, Japan). Samples resuspended in 20 mM Tris-HCl buffer, pH 8.0 (10 to 20 μg · μl^-1^), were mixed in a 1:1 ratio with matrix Super dihydroxybenzoic acid (Super DHB), and 1 μl of this mixture was loaded on a DE1580TA plate (from Shimadzu) and allowed to dry at room temperature. Super DHB was prepared from 9 parts 2,5-dihydroxybenzoic acid (DHB) and 1 part 2-hydroxy-5-methoxybenzoic acid (Sigma-Aldrich, St. Louis, MO), both prepared in 70% acetonitrile–29.9% H_2_O–0.1% trifluoroacetic acid. NMR experiments were performed on a Bruker Advance 700 (Bruker, Allentown, PA) with a Bruker 5-mm TCI 700 H/C/N cryoprobe or on a Bruker Advance 800 with a Bruker 5-mm TCI 800 H/C/N cryoprobe. NMR solutions were made using15 to 40 mg uniformly ^15^N-MB-OB3b in a 90:10 H_2_O-D_2_O mixture at pH 6.5. Unless otherwise specified, all experiments were run at 265 K and 300,000,000 Pa. Samples were placed in 300,000,000-Pa-rated sapphire NMR tubes (Daedalus Innovations, LLC, Beaverdam, VA), and high pressure was generated by an Xtreme 60 (Daedalus Innovations). Analysis was performed in Mnova (Mestrelab Research, Escondido, CA).
