# Supplementary material for: MbnC Is Not Required for the Formation of the N-Terminal Oxazolone in the Methanobactin from Methylosinus trichosporium OB3b
Source: Appl Environ Microbiol. 2022 Jan 25;88(2):e01841-21. doi: 10.1128/AEM.01841-21 (PMC8788703; doi:10.1128/AEM.01841-21)
Supplement: Supplemental file 1 — Fig. S1 to S8. Download AEM.01841-21-s0001.pdf, PDF file, 1.9 MB [file aem.01841-21-s0001.pdf]

Revised manuscript: AEM01841-21

Supplemental information

Mbn C is required for the formation of the C-terminal oxazolone, but not for the formation of the N-terminal oxazolone in methanobactin from *Methylosinus trichosporium* OB3b.

Philip Dershwitz[1,Ψ], Wenyu Gu[2Ψ‡], Julien Roche[1], Christina S. Kang-Yun[2†],Jeremy D. Semrau[2], Thomas A. Bobik[1], Hans Zischka[3], and Alan A. DiSpirito[1]*

Supplemental Figures

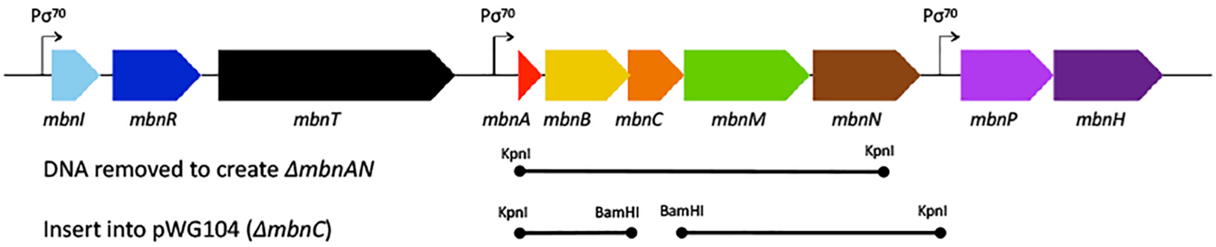

Fig. S1. Methanobactin gene cluster of *M. trichosporium* OB3b. DNA removed/added to construct *ΔmbnAN* and *ΔmbnC* mutants indicated below the gene cluster.

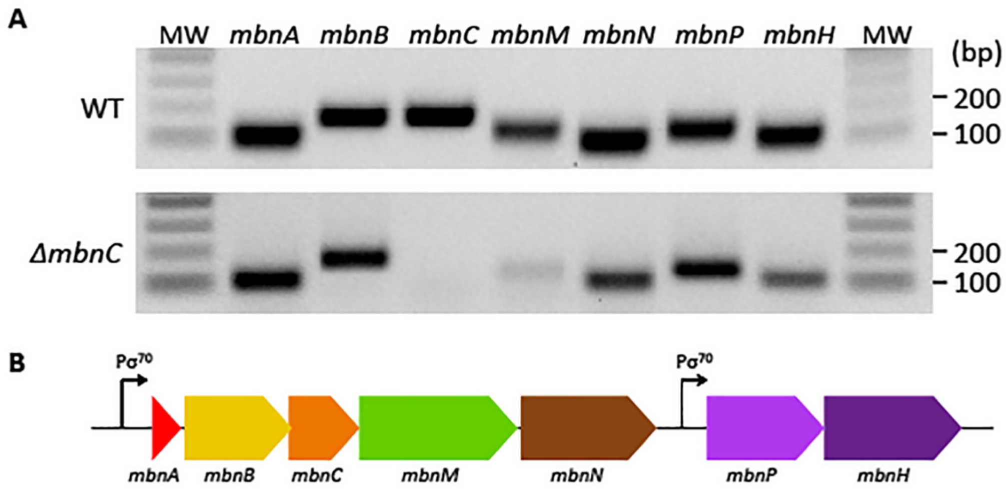

Figure S2. Expression of *mbnABCMNPH* in *M. trichosporium* OB3b wildtype (WT) and Δ*mbnC* grown with 0.2 µM Cu as *confirmed* via RT-PCR. (A). *mbn* biosynthesis genes in *M. trichosporium* OB3b are shown in (B). MW – molecular weight marker.

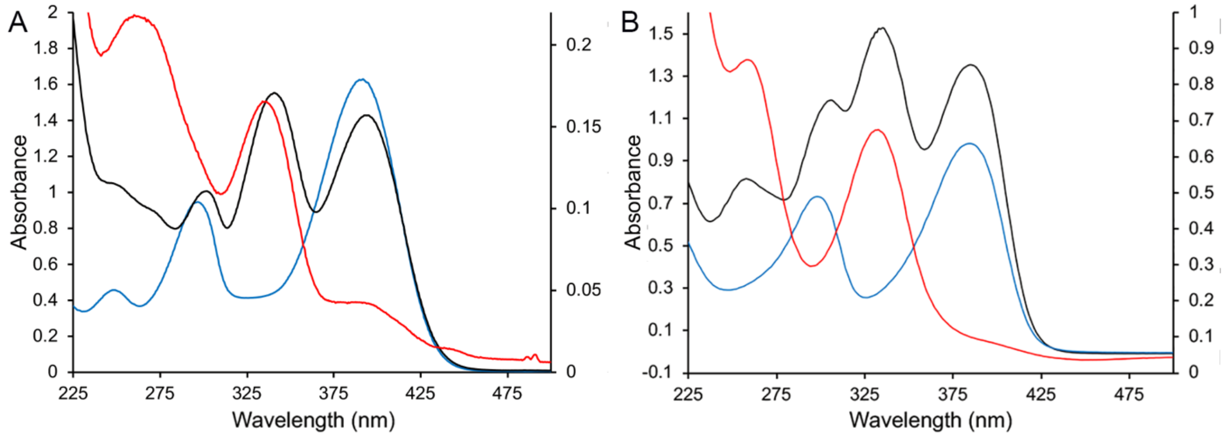

Fig. S3. HPLC fractions of from freeze dried Dianion HP-20 samples from the spent media of M. *trichosporium* OB3b (A) and the Dianion HP-20 samples from the spent media of *Methylocystis* strain SB2 (B). (A) Holo-MB-OB3b fraction (black curve), MB-OB3b minus the C-terminal oxazolone group fraction (blue trace) and MB-OB3b minus the N-terminal oxazolone group (red trace). (B) Holo-MB-SB2 fraction (black trace), MB-SB2

minus imidazolone/pyranzadione fraction (red trace), and MB-SB2 minus oxazolone fraction (red trace).

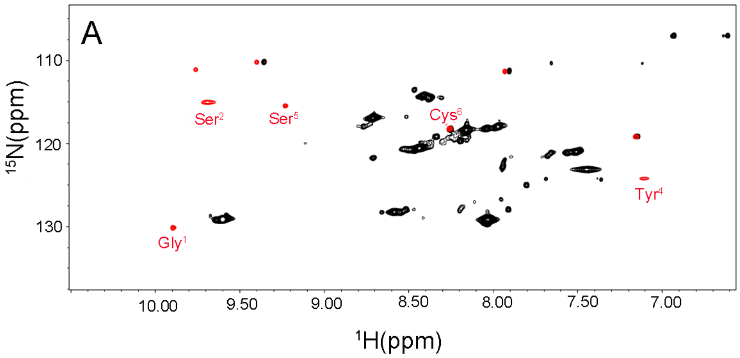

Fig. S4. (A) 700 MHz 9^{1}H,^{15}N-HSQC spectrum of uniformly ^{15}N-labeled MB from wild type MB-OB3b (black) and following the addition of equimolar Cu^{2+} (red)$^-$ in 90% 9 mM phosphate buffer, pH 6.5, and 10% D_2O at 298K and 1 bar.

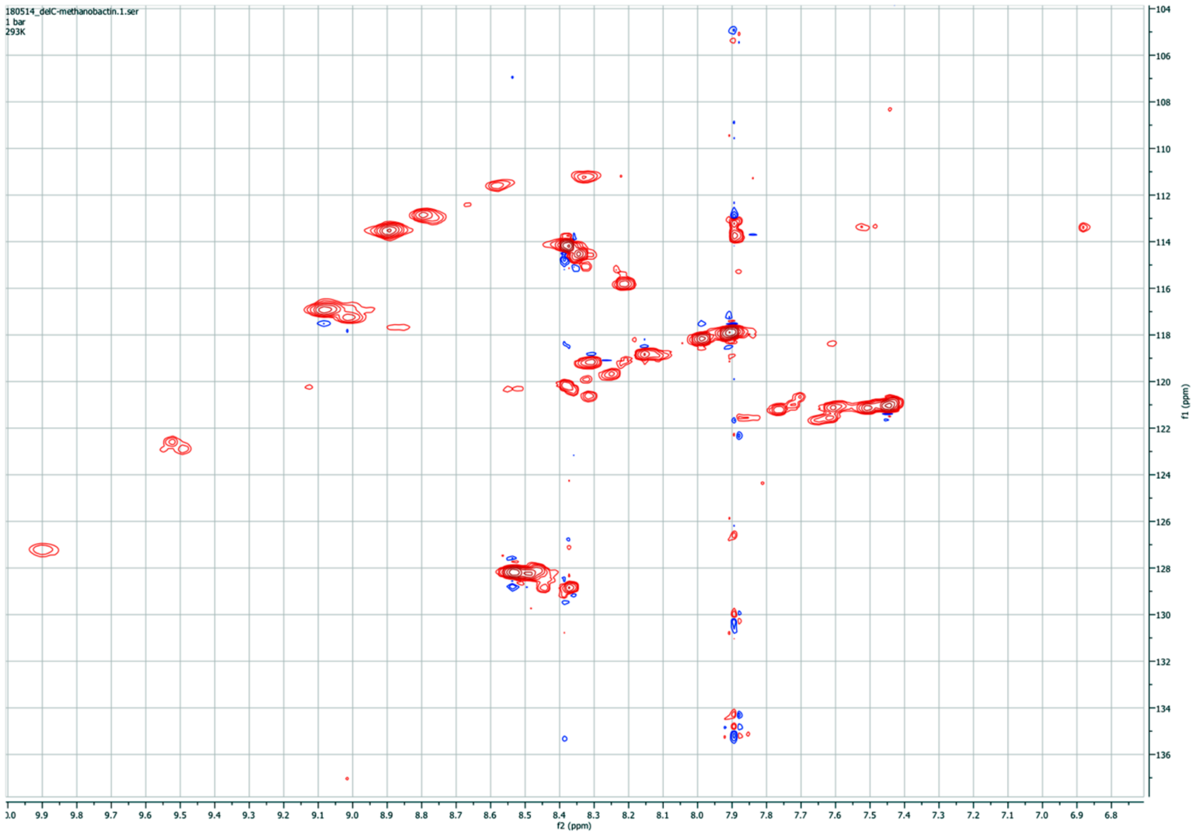

Fig. S5. 800 MHz (1H,15N)-HSQC spectrum of uniformly 15N-labeled **Δ**MbnC in 90% 9 mM phosphate buffer, pH 6.5, and 10% D_2O at 293K and 1 atm. Positive component is in red, negative component is in blue.

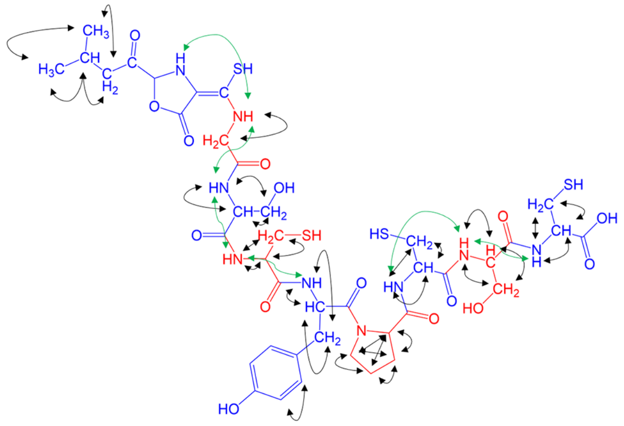

Fig. S6. TOCSY (black) and ROESY (green) correlations observed in 2D NMR for ΔMbnC.

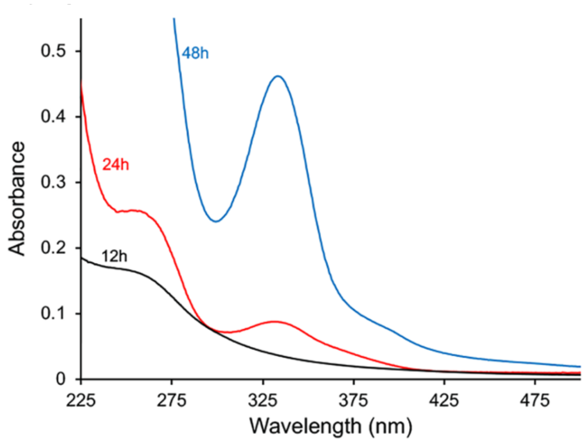

Fig. S7. Spent media from *ΔmbnN* strain of *M. trichosporium* OB3b. Samples were taken 12h (black trace), 24h (red trace) and 48h (blue trace) following inoculation. Culture samples were centrifuged at 12,000 x g for 20 min, filtered through 0.2µm Millipore filter and assayed by UV-visible absorption spectra.

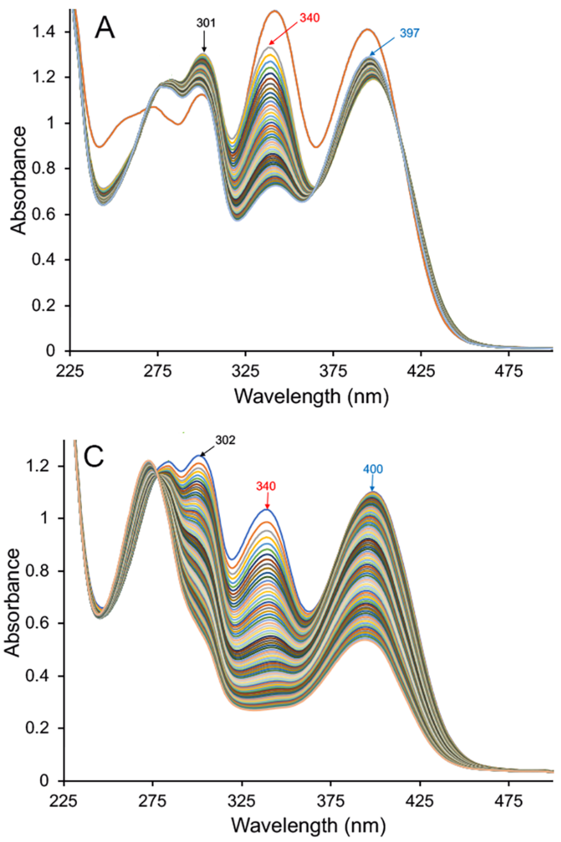

Fig. S8. Acid hydrolysis of the group I methanobactin *M. parvus* OBBP (1). (A and C) Spectral changes in the absorbance spectra of the MB from M. parvas OBBP in 85µM acetic acid. (B and D) Absorbance changes at 302nm (black circles), at 340 nm (red circles) and at 400nm over time.

(2)

Citations

1. DiSpirito AA, Semaru JD, Murrell JC, Gallagher WH, Dennison C, Vuilleumier S. 2016. Methanobactin and the link between copper and bacterial methane oxidation. Microbiol Mol Biol Rev 80:387-409.

2. Semau JD, DiSpirito AA, Obulisamy PK, Kang CS. 2020. Methanobactin from methanotrophs: genetics, structure, function and potential applications. FEMS Microbiol Lett 367:feaa045.
